# Self-perceived competencies on evidence-based medicine in medical students and physicians registered in a virtual course: a cross-sectional study

**DOI:** 10.1080/10872981.2021.2010298

**Published:** 2021-12-17

**Authors:** Milton A. Romero-Robles, David R. Soriano-Moreno, Fabrizio M. García-Gutiérrez, I. Benjamín Condori-Meza, Caroline C. Sing-Sánchez, Sandy P. Bulnes Alvarez, Christoper A. Alarcon-Ruiz, Alvaro Taype-Rondan, Andres Viteri‐García

**Affiliations:** a Escuela de Medicina, Universidad Nacional Del Santa, Nuevo Chimbote, Ancash, Perú; b Comité Permanente Académico, Sociedad Científica Médico Estudiantil Peruana, Lima, Perú; c Escuela de Medicina, Universidad Peruana Unión, Lima, Perú; d Escuela de Medicina, Universidad Nacional de Trujillo, Trujillo, Perú; e Escuela de Medicina, Universidad César Vallejo, Piura, Perú; f Unidad de Investigación Clínica y Epidemiológica, Escuela de Medicina, Universidad Peruana Unión, Lima, Perú; g Centro de Investigación de Salud Pública Y Epidemiología Clínica (Cispec). Facultad de Ciencias de La Salud Eugenio Espejo, Universidad Ute, Quito, Ecuador; h Fundación Epistemonikos, Santiago, Chile

**Keywords:** Evidence-based medicine, physicians, professional competence, Medical students, Medical education

## Abstract

**Background:**

Evidence-based medicine (EBM) is defined as the integration of the best available evidence from scientific studies with clinical experience (and context) and with patients’ values and preferences. The objective of the present study was to describe self-perceived EBM competencies in physicians and medical students enrolled in a massive virtual EBM course.

**Methods:**

Analytical cross-sectional study. People interested in a free virtual EBM course fulfilled their data in a virtual form for their registration in September 2020. In this form, 22 competencies related to four dimensions of EBM were evaluated: asking a clinical question, search, analysis, and application; using a 5-option Likert scale. The resulting database was analyzed, selecting people who claimed to be physicians or medical students of 18 years or more.

**Results:**

1793 participants were included: 1130 medical students and 663 physicians; more than 80% lived in Peru. The frequency of participants who agreed or strongly agreed with feeling qualified in each competence ranged: from 39.2% to 57.8% for the competencies of the ‘Asking a clinical question’ dimension, from 39.2% to 56.1% for ‘Search,’ from 19.9% to 32.0% for ‘Analysis,’ and from 19.6% to 29.9% for ‘Application.’ Both in physicians and students, the lowest frequencies were for the competencies of interpretation of impact measures, graphs, and results of systematic reviews; as well as shared decision making and calculation of expected benefit. Physicians who graduated more recently scored better on competencies from search and analysis dimensions.

**Conclusion:**

Among physicians and medical students enrolled in the course, self-perception of competencies was lower in the dimensions of analysis and application. More recently graduated physicians seem to have a greater self-perception of their research and analysis skills, probably due to curricular updates.

**List of abbreviations:** EBM: Evidence-based medicine; CIMBE, for its acronym in Spanish: International Course on Evidence-Based Medicine; SOCIMEP, for its acronym in Spanish: Peruvian Medical Student Scientific Society

## Introduction

Evidence-based medicine (EBM) is defined as the integration of the best available evidence from scientific studies with clinical experience (and context) and with patients’ values and preferences [1]. The objective of EBM is to help make rational decisions for the patient’s benefit [2–4]. There are five steps for the EBM process: to ask a clinical question (formulating a clinical question), search (looking for scientific studies that answer the question), analyze (critically reading the studies), application (applying the evidence to make a decision), and evaluation (assessing the decision made) [5].

The use of EBM has barriers such as lack of digital skills, low access to online information, lack of time, the science-practice gap [6], and lack of competencies among health personnel [7,8]. Accordingly, previous studies in Peru [9–11] and other countries [12–15] have found that physicians and medical students often have conceptual and procedural shortcomings regarding EBM competencies. However, these studies focus on assessing a few competencies (usually literature search and interpretation of measures of association) without exploring other competencies relevant to evidence-based decision making.

The scarcity of studies on this subject makes it difficult to know in which competencies physicians and medical students may have more deficiencies, and therefore the design of interventions in this area [16]. Thus, the objective of the present study was to describe self-perceived EBM competencies in physicians and medical students enrolled in a massive virtual EBM course.

## Materials and methods

### Study design, context, and objective

This was a cross-sectional study that analyzed a database obtained from the registration to a free virtual course called ‘International Course on Evidence-Based Medicine’ (CIMBE, for its acronym in Spanish), organized by the ‘Peruvian Medical Student Scientific Society’ (SOCIMEP, for its acronym in Spanish), whose objective is to carry out academic-scientific activities that promote health research at the undergraduate level [17]. The research team, formed by medical students and health professionals with training and experience in EBM, were also organizers and faculty for the CIMBE course.

### Participants

The CIMBE was a free course available to Spanish-speaking students and health professionals (physicians, nurses, obstetricians, dentists, nutritionists, etc.). To register for the course, those interested had to fill out a form. Only those enrolled in the course who described themselves as physicians or medical students were included in the present study. Those under 18 years of age and those who did not complete the variables of interest (EBM competencies) were excluded.

### Procedures and variables

The dissemination of the CIMBE was made from 5th to 19 September 2020, through the official social networks of SOCIMEP (on Facebook, Twitter, and Instagram) and in study groups to which the course organizers belonged, on WhatsApp and Telegram. In addition, invitation emails were sent to the following institutions: Epistemonikos and Cochrane associated centers from Chile, Mexico, Ecuador, and Argentina; whose members also participated as speakers in the course.

The course registration was performed from September 5th to 26th, 2020 through Google Forms. This form had two parts. The first part included general data on the participants such as age, sex, country of residence, profession studied or currently studying, current academic degree, whether they were undergraduate students, current year of study or year of graduation, whether they have a master’s or doctoral degree, whether they belong or belonged to a student scientific society and the self-reported number of articles published in scientific journals.

The second part of the form was an instrument created to evaluate the self-perceived competencies in EBM in health care students and professionals. The development of this instrument was based on the essential competencies in evidence-based practice for health professionals, which were defined by consensus in a previous study [3]. This study established 27 competencies divided into six dimensions: introduction (5 competencies), asking a clinical question (3 competencies), search (4 competencies), analysis (9 competencies), application (4 competencies), and evaluation (2 competencies). This instrument was originally developed to obtain baseline data on course participants.

For the development of the instrument, we included all the competencies of asking a clinical question, search, analysis, and application dimensions (20 competencies in total). The introduction and evaluation dimensions were not included in the instrument because we considered that their competencies were unspecific and difficult to operationalize in a questionnaire that evaluated self-perception.

Questions were asked to assess whether participants feel able to perform each of the competencies on their own, with responses on a Likert scale of five categories (1 = strongly disagree, 2 = disagree, 3 = neither agree nor disagree, 4 = agree, and 5 = strongly agree). For this purpose, the phrasing of the competencies of the reference mentioned above was kept verbatim.

Each competency was independently asked by one question, except for the second competency of the analysis dimension (competency 3.2: ‘interpret different types of measures of association and effect, including graphical presentations’). This competence was divided into three questions (evaluating: measures of association, measures of impact, and graphs). Therefore, a total of 22 questions were formulated. Finally, the frequencies of having responded ‘agree’ or ‘strongly agree’ with feeling qualified to perform each competency were calculated, and the average score for each competency was calculated. Also, we asked whether during undergraduate studies the participant had taken classes in which each of the dimensions mentioned in the form was addressed.

A preliminary test of the form was carried out on a group of 10 students and 10 health professionals to check whether the questions were understood. The members of the course organizing team and their advisors discussed the doubts and suggestions obtained in this test, according to which the questions were reformulated.

### Statistical analysis

The database was imported into the Stata/SE version 14 (StataCorp, Texas, USA) statistical software. Before statistical analysis, we evaluated whether there were duplicate records, taking into account the coincidence of surnames and names, and then proceeded to anonymize the database. Then, we searched for possible implausible data in the age variable and the number of articles published. If they were considered not plausible, they were considered as missing data.

For the descriptive analysis, absolute and relative frequencies were used, as well as measures of central tendency and dispersion. For the subgroup of physicians, we considered that due to the changes in the curricula regarding research and EBM during the past years, the perceived competencies could be higher in those recently graduated (who were exposed to a newer curriculum) than in those that graduated earlier. In order to test this hypothesis, we assessed the association between the year of graduation (in tertiles) and the mean score for each competencies dimension. For this, we calculated the coefficients (β) and their 95% confidence intervals (95% CI) using simple linear regression with robust variance, as well as a mixed-model linear regression adjusted for sex, master degree, doctor degree, having belonged to a students scientific society, and having published papers; with the country as a random effect.

### Ethical aspects

The present study was approved by the Ethics Committee of the Universidad Peruana Union (Code: 2020-CEUPeU-00028). The database to be used did not collect sensitive information from the participants and was anonymized before starting data processing. It was not possible that individuals could be identified based upon any demographic item. The authors were granted permission to use the course participants’ registry database by SOCIMEP.

## Results

After deduplication, data were collected from 2325 persons enrolled in CIMBE. 425 were excluded because they were not physicians or medical students, one because they were under 18 years of age, seven because they did not provide their academic degree, and 99 because they did not provide data on their EBM competencies. Finally, data from 1793 medical students or physicians enrolled in the CIMBE were analyzed.

Of these 1793 individuals, 663 (37.0%) were physicians and 1130 (63.0%) were medical students. The median age was 23 years old in medical students and 30 years old in physicians. Regarding the gender of the participants, 63.9% of the medical students and 45.7% of the physicians were female. More than 80% of students and physicians reported living in Peru. Regarding scientific publications, 13.9% of the students and 30.0% of the physicians reported that they had published at least a scientific article (Table 1).

**Table 1.** Characteristics of the physicians and medical students enrolled in the CIMBE (n = 1793)

Variable	Medical studentsn (%)	Physiciansn (%)
Age: median (IQR)*	23 [21–25]	30 [27–37]
Gender: Female	722 (63.9)	303 (45.7)
Country:		
Peru	915 (81.0)	556 (83.9)
Mexico	44 (3.9)	15 (2.3)
Bolivia	67 (5.9)	40 (6.0)
Others	104 (9.2)	52 (7.8)
Number of publications:		
0	973 (86.1)	464 (70.0)
1	94 (8.3)	104 (15.7)
2	36 (3.2)	43 (6.5)
3 or more	27 (2.4)	52 (7.8)
Has belonged or belongs to a student scientific society	668 (59.1)	177 (26.7)
Report having a master’s degree		66 (10.0)
Report having a doctor’s degree		15 (2.3)

IQR: interquartile range

Regarding self-perceived competence in EBM, the frequency of participants who agreed or strongly agreed that they felt trained in each competency ranged from 39.2% to 57.8% for the competencies of ‘Asking a clinical question’ dimension, from 39.2% to 56.1% for ‘Searching’, from 19.9% to 32.0% for ‘Analysis’, and from 19.6% to 29.9% for ‘Application’. In both physicians and medical students, the lowest frequencies were found for the competencies of interpretation of impact measures, graphs, and results of systematic reviews, as well as for results of systematic reviews; as well as shared decision making and calculation of expected benefit (Table 2).

**Table 2.** Percentage of participants who agree or strongly agree with feeling trained to carry out each of the activities described (in = 1793)

Competencies	All (medical students or physicians)(n = 1793)n (%)	Physicians (n = 663)n (%)
**Dimension 1: Question**		
1.1) Differentiate which questions can be answered by research studies, and searches cannot.	945 (52.7)	360 (54.3)
1.2) Identify the different types of clinical questions (questions about treatment, diagnosis, prognosis, and etiology).	1036 (57.8)	411 (62.0)
1.3) Convert a clinical question into a question in PICO format or its variants when appropriate (PICOT, PECO, PO).	703 (39.2)	245 (37.0)
**Dimension 2: Search**		
2.1) Explain the results are the main databases and other resources (guides, reviews, UpToDate, others) to search for health evidence.	960 (53.5)	334 (50.4)
2.2) Build and carry out a suitable search strategy for clinical questions (including Boolean operators, truncation, and/or filters).	703 (39.2)	260 (39.2)
2.3) Identify the differences between the main databases of scientific information.	775 (43.2)	281 (42.4)
2.4) Obtain the full text of the scientific articles and/or resources, as needed.	1005 (56.1)	351 (52.9)
**Dimension 3: Analysis**		
3.1) Identify how reliable a study is based on its biases, confidence intervals, confounders, conflicts of interest, and subgroup analysis.	529 (29.5)	217 (32.7)
3.2) Interpret the different measures of association (PR, OR, RR, HR, MD).	526 (29.3)	229 (34.5)
3.2) Interpret the different impact measures (RA, NNT, and NNH).	377 (21.0)	170 (25.6)
3.2) Interpret the graphs most used in clinical studies (Kaplan-Meier, cumulative incidence).	357 (19.9)	162 (24.4)
3.3) Value and interpret critically a systematic review, meta-analysis, forest plot and summary of findings table (SoF).	369 (20.6)	165 (24.9)
3.4) Value and interpret critically a treatment study (randomized clinical trial), as well as an observational study with statistical adjustment.	433 (24.1)	186 (28.1)
3.5) Value and interpret critically a diagnostic precision study (a study that presents sensitivity and specificity).	481 (26.8)	210 (31.7)
3.6) Distinguish between evidence-based clinical practice guidelines (based on systematic reviews), and opinion-based guidelines.	574 (32.0)	245 (37.0)
3.7) Identify the key features of a prognostic study (clinical prediction study) and be able to interpret it.	413 (23.0)	185 (27.9)
3.8) Explain in which cases to explain harm from interventions I rely on clinical trials and in which cases I rely on observational studies	412 (23.0)	177 (26.7)
3.9) Explain the purpose and processes of a qualitative study and how it can be used to make decisions.	479 (26.7)	195 (29.4)
**Dimension 4: Application**		
4.1) Carry out a shared decision-making process (*shared decision making*) with the patient, including explaining the evidence (*decision aids*) to the patient and integrating their preferences.	371 (20.7)	194 (29.3)
4.2) Recognize the components and professional, ethical, and legal dimensions of clinical decision-making and the role of clinical reasoning.	537 (29.9)	245 (37.0)
4.3) Calculate the individual expected benefit of a certain intervention based on my patient’s initial risk (in terms of AR or NNT).	352 (19.6)	172 (25.9)
4.4) Interpret the certainty in the evidence (GRADE methodology) and the strength of the recommendations in health care.	402 (22.4)	199 (30.0)

Out of the 663 physicians enrolled in the CIMBE, 632 recorded their year of graduation. In these, the association between the year of graduation and the average self-perception score in each dimension of competencies (obtained by averaging the scores of all the items corresponding to the dimension) was evaluated. We found that for the dimensions ‘Asking a clinical question’, ‘Searching’, and ‘Analysis’, those who graduated more recently presented higher average scores (Table 3).

**Table 3.** Association between graduation year (in tertiles) and mean score in each dimension, in physicians enrolled in the CIMBE (n = 632)*

Graduation Date	Score ± standard deviation *	Crude β (IC 95%)	Adjusted β (IC 95%) **
Dimension 1: Question			
1981 to 2014	3.22 ± 1.00	Ref	Ref
2015 to 2018	3.17 ± 1.01	−0.05 (−0.24 to 0.14)	0.01 (−0.22 to 0.25)
2019 to 2020	3.34 ± 0.94	0.11 (−0.07 to 0.30)	**0.18 (0.04 to 0.33)**
Dimension 2: Search			
1981 to 2014	3.04 ± 0.96	Ref	Ref
2015 to 2018	3.17 ± 1.02	0.13 (−0.06 to 0.32)	0.18 (−0.08 to 0.44)
2019 to 2020	3.30 ± 0.97	**0.26 (0.07** to **0.44)**	**0.32 (0.19** to **0.44)**
Dimension 3: Analysis			
1981 to 2014	2.73 ± 0.90	Ref	Ref
2015 to 2018	2.89 ± 0.90	0.16 (−0.02 to 0.33)	0.19 (−0.07 to 0.45)
2019 to 2020	2.93 ± 0.90	**0.20 (0.03** to **0.37)**	**0.23 (0.06** to **0.40)**
Dimension 4: Application			
1981 to 2014	2.83 ± 0.91	Ref	Ref
2015 to 2018	2.84 ± 0.97	0.01 (−0.17 to 0.20)	0.04 (−0.09 to 0.17)
2019 to 2020	2.88 ± 0.95	0.05 (−0.13 to 0.23)	0.09 (−0.07 to 0.25)

*The scores are the averages for the items in each dimension, which had 5 categories (from totally disagree [1 point] to totally agree [5 points])**Mixed-model linear regression adjusted for sex, master degree, doctor degree, having belonged to a students scientific society, and having published papers; with the country as a random effect

## Discussion

### Summary of findings

The present study was conducted in a sample of physicians and medical students enrolled in an EBM course. Their self-perceived competencies in EBM were generally better for asking a clinical question and searching for evidence, compared to competencies in critical analysis and applying evidence to make decisions. Also, physicians who graduated longer ago were less likely to perceive themselves as having competencies in the search and analysis dimensions compared to those who graduated less time ago.

### Comparison with previous studies

In the present study, an exhaustive list of EBM competencies has been used for the assessment of self-perception, whereas previous studies have focused on certain specific competencies. A systematic review of studies assessing EBM competencies [18] found that the instrument most commonly used by previous studies was the McColl questionnaire [13]. This questionnaire focuses only on assessing knowledge of databases, interpretation of statistical results, and concepts of systematic reviews. Other questionnaires used in previous studies [12,14,15,19,20] also focus on some specific statistical and methodological topics. Likewise, studies that have assessed competencies in the form of a graded test [8,21–24] have focused on certain basic methodological concepts such as the definition of EBM, the hierarchy of evidence, and the interpretation of statistical results.

The systematic review above [18] included 57 studies and reported that on average about 60% of physicians reported a high level of knowledge of EBM concepts. However, because these studies have only evaluated some specific competencies and to the great heterogeneity of the instruments used, likely, this result does not reflect the full range of competencies required to apply EBM in medical practice. Therefore, it is important to carry out studies with a more exhaustive approach to these competencies, allowing their evaluation.

We found that the competencies with the highest self-perception were those belonging to the dimensions of asking a clinical question and searching for evidence, and those with the lowest self-perception were those of critical analysis of the evidence and application of the evidence to make a decision. Regarding the competency of asking a clinical question, a study conducted among physicians in Mexico found that 55% of the participants were aware that the first phase of EBM is the formulation of the clinical question, but did not evaluate this competency in more depth [25]. In the present study, a similar frequency was found for the competencies of identifying a clinical question; however, this decreased when asked about the competency of formulating a clinical question in PICO or a similar format. Concerning the search dimension, a study conducted on physicians in training in gynecology in the UK revealed that 35% had minimal literature search skills [21]. Similar to the previous dimension, we reported that half of the respondents were aware of databases where to search and obtain scientific articles, however, self-perceived competence to design and perform a literature search decreased by about 39%.

Regarding the ‘analysis’ dimension, a systematic review conducted in 2017 found 57 studies. In that study, authors reported that between 17.5% and 92% of physicians had some knowledge of the concepts of systematic reviews and meta-analyses and between 8.7% and 95% for biostatistics concepts [18]. These results are very imprecise and difficult to compare with the present investigation since they have not evaluated concepts such as attributable risk, the number needed to treat, or the interpretation of the most commonly used graphs in clinical studies [26,27]. For the application dimension, we did not find previous studies that have evaluated this dimension from the approach proposed by EBM. What stands out from our results is that almost all the competencies evaluated in these two dimensions do not exceed 30%. This is probably because the isolated non-formal training in EBM in recent years has prioritized the questioning and searching dimensions. However, we recognize that adequate competencies in these dimensions alone will not be sufficient to apply EBM well. So, training and assessing competence such as understanding research results, evidence summaries, and their application with each patient according to his/her preferences should be also prioritized [28].

One of the challenges for medical education in Peru is the training in EBM [29]. During the last few years, extracurricular courses have been performed in several universities, and three universities implemented an EBM course into their curricula. However, these courses are mainly focused on theoretical background and application of the three first steps of EBM. This could explain why more recently graduated Peruvian physicians have higher self-perceived competencies for search and analysis than those graduated before. This is contrary to a study conducted in Sri Lankan, where specialist physicians had the best competencies concerning EBM and the highest use of EBM compared to general practitioners [25,30]. So, Peruvian physicians with more professional experience may prefer to be guided by clinical experience or the opinion of their colleagues rather than by EBM, because of their lack of exposure to any formal or informal training in EBM [31].

EBM training is mainly focused on theoretical courses and workshops, especially in the first steps of EBM [32,33]. Some educational interventions showed increased knowledge, skills, and willingness to apply EBM in clinical practice [34–36]. However, little is known regarding which are the best interventions to reach an evidence-based practice, and how context-dependent they are [37].

Our results suggest that a large proportion of the respondents perceive that they do not have the necessary skills to perform the analysis, application, and evaluation steps of EBM. This situation could lead to poor evidence-based decision-making in Peru, as seen during the COVID-19 pandemic [38,39]. Despite public institutions in the country having initiatives to make evidence-based decisions [40,41], there is still a huge need to establish structural interventions, which may include institutional motivation, an environment that promotes EBM application, and formal theoretical and practice training in EBM [42]. This last element may be adapted to some populations’ specific needs according to their specific previous experience and training in EBM. So, before implementing training in EBM, it may be important to assess the base knowledge and competences of the target population to be trained.

### Strengths and limitations

Some important limitations should be kept in mind when interpreting the results of the present study: 1) The study assessed self-perception of competencies, and not practice in real scenarios. Although self-perception may underestimate or overestimate the real competencies of medical students and physicians, its evaluation is important, understanding that a high self-perception in a certain activity would be related to a greater willingness to perform that activity [43]. 2) Since this study was carried out on persons enrolled in a virtual EBM course, the extrapolation of the results obtained should be done with caution, since it’s possible that the persons enrolled in the course were those with a greater interest in the subject, who have already made previous efforts to acquire competencies in EBM, and therefore the competencies found in this group could overestimate those of the rest of the physicians and medical students. However, it is also possible that the opposite may have occurred: that those professionals and students who already believe they have competencies in this regard may have considered it of little relevance to enroll in the course. Therefore, we did not seek to extrapolate the descriptive results but rather focus on which competencies the participants feel less skilled. 3) The population of physicians included in our study has a large range of years of professional practice (from 1981 to 2020), so it is not a homogeneous population, as previous experience in clinical practice or research may affect their self-perception of their EBM competencies.

Despite its limitations, the present study is, to our knowledge, the largest population-based study that has assessed self-perceptions of EBM competencies in physicians or medical students in Latin America. Besides, it has evaluated an exhaustive list of competencies, covering the spectrum required for the adequate practice of EBM.

## Conclusion

In conclusion, medical students and physicians have a low self-perception of having acquired competencies to perform EBM, especially in the dimensions of evidence analysis and application of evidence to make decisions. Particularly, physicians who graduated longer ago would have an even lower self-perception of competencies. Medical students and physicians require theoretical and practice training in EBM.

## Data Availability

The datasets used and/or analyzed during the current study are available from the corresponding author on reasonable request.
